# Large hiatus hernia: time for a paradigm shift?

**DOI:** 10.1186/s12893-022-01705-w

**Published:** 2022-07-08

**Authors:** Kheman Rajkomar, Christophe R. Berney

**Affiliations:** Bankstown-Lidcombe Hospital, Sydney, Australia

**Keywords:** Hiatal hernia, Paraesophageal hiatal hernia, Surgical mesh, Laparoscopy

## Abstract

**Background:**

Laparoscopic large hiatal hernia (LHH) repair remains a challenge despite three decades of ongoing attempts at improving surgical outcome. Its rarity and complexity, coupled with suboptimal initial approach that is usually best suited for small symptomatic herniae have contributed to unacceptable higher failure rates.

**Results:**

We have therefore undertaken a systematic appraisal of LHH with a view to clear out our misunderstandings of this entity and to address dogmatic practices that may have contributed to poor outcomes.

**Conclusions:**

First, we propose strict criteria to define nomenclature in LHH and discuss ways of subcategorising them. Next, we discuss preoperative workup strategies, paying particular attention to any relevant often atypical symptoms, indications for surgery, timing of surgery, role of surgery in the elderly and emphasizing the key role of a preoperative CT imaging in evaluating the mediastinum. Some key dissection methods are then discussed with respect to approach to the mediastinal sac, techniques to avoid/deal with pleural breach and rationale to avoid Collis gastroplasty. The issues pertaining to the repair phase are also discussed by evaluating the merits of the cruroplasty, fundoplication types and gastropexy. We end up debating the role of mesh reinforcement and assess the evidence with regards to recurrence, reoperation rate, complications, esophageal dilatation, delayed gastric emptying and mortality. Lastly, we propose a rationale for routine postoperative investigations.

## Introduction

The history of the hiatus hernia and its repair is rich and eventful. It took nearly half of a century between its first description by Bowditch in 1853 and a reported case of elective open repair by Soresi in 1919. About seven decades later the first laparoscopic hiatal hernia repair was undertaken by Dallemagne in 1991. Shortly after, in 1992, Cusheri performed a repair for a large hiatus hernia, and a year later mesh reinforcement of laparoscopic paraesophageal hernia was described. Over the last three decades the laparoscopic approach to large hiatal/paraesophageal hernia repair has become increasingly ubiquitous.

However, whilst repair of small symptomatic sliding hiatal herniae has been well described and has yielded good outcomes the same cannot be said of large hiatal herniae (LHH). Its rarity and complexity, coupled with suboptimal technical approach that is usually best suited for small defects have contributed to unacceptably high failure rates. Moreover, the range of confounders present in published series on LHH makes it difficult to reach any meaningful conclusion and also to draw relevant comparisons across various series. The absence of good quality evidence has resulted in lack of clarity in EAES and SAGES guidelines, thus leaving the decision to the individual surgeons. It is therefore not surprising that a survey of international surgeons has shown wide variations of techniques and use of mesh [1, 2].

It is time to address innumerable unanswered controversies related to this topic as, with improvement in life expectancy and better management of comorbidities, bigger numbers of LHHs occurring in an aging population are bound to be repaired. In fact, the proportion of LHH that makes up the caseload of a specialised unit in Australia has increased from 10 to 30% [3]. An intensified workload of such complex benign surgery in elderly patients has the potential for a corresponding increased rate of morbidity and mortality, with an inherent associated cost, if evidence-based practice remains suboptimal. We therefore propose a systematic appraisal of LHH, stressing on definition of terms, preoperative assessment, techniques of dissection and repair, and postoperative care. We pay particular attention in deconstructing some dogmas (such as use of Collis gastroplasty) that has infiltrated surgical practice due to the void caused by lack of good quality data and may have contributed to poor outcomes.

## Definition of terms

Whilst hiatal herniae (HH) are defined as per the Hill classification, there are no clear well-defined terms to categorise what a large hernia really means. Various terms such as intrathoracic, giant and complex hernia are often used loosely or interchangeably but without properly defined objective measurement. Our aim is to propose a more reliable and practical classification method for such large HH.

Large HH can be subclassified based on the volume of stomach in the thoracic cavity or on the size of the hiatus. Intrathoracic stomach is defined as a hernia with at least a third of the stomach in the chest [4], whereas a giant hernia demonstrates > 30–50% of the stomach incarcerated in the mediastinum. They mostly include Hill type 3 and 4 herniae [5]. More complex type 4 herniae contain stomach and additional viscera e.g. colon, spleen, pancreas. At laparoscopy the volume of stomach herniating into the chest can be determined by looking at the part of the stomach that is found at the level of the crura. The pylorus, crow’s foot or a point halfway between the crow’s foot and the angle of His at the crura usually correspond to 100, 75 or 50% of the stomach having herniated respectively [6].

Crural defect size can be more directly and objectively measured with tape or using graspers as surrogate of size. Champion considered 5 cm as being the threshold for the hernia to be deemed large [7]. Alternatively the area created by the crural defect can be calculated by measuring the radius of the defect and the size of the arc [8]. The surface area 10–20 cm^2^ is considered large and > 20 cm^2^ is termed huge or giant [9].

Accurate description of the hernia by size is important when investigating outcomes by hernia size. Very often herniae are deemed large subjectively rather objectively. Also, the appropriate classification of the hernia helps to prognosticate recurrence as often recurrence is size dependent. We propose that a sliding hiatus hernia ≥ 7 cm and/or ≥ 50% of the stomach having herniated through the crura being considered LHH.

## Rationale for operating

### Evolution from routine to selective operating in patients

In the twentieth century most large, even asymptomatic, paraesophageal herniae (PEH) were repaired in order to avoid the risk of complications which could lead to the patient’s demise. This was borne out of previous reports that quoted a mortality rate of 26% should patients present on emergency with complications from their herniae, compared to reduced mortality of 2% when those herniae are repaired electively [10, 11].

However, a Markov analysis in 2002 did put the risk of non-intervention into perspective. Essentially, patients older than 65 years with no or minimal symptoms from their hernia had a 1.16% risk per year of needing an urgent operation [12]. Thus, the lifetime risk reduces significantly from 18% for a 65-years-old to 7% for an octogenarian. A subsequent similar analysis showed an improvement in Quality-of-Life (QoL) expectancy by 5 months with non-operative approach for those patients [13]. A 10 year audit of the National Inpatient Sample database in US showed only 0.9% admission rate for complicated PEH [14]. Hence watchful waiting has been deemed more appropriate for minimally symptomatic and older patients.

### Elicit both typical and atypical symptoms when evaluating LHH

Marked deformity around the gastroesophageal junction can lead to typical symptoms of reflux and dysphagia. A disruption of the phrenoesophageal ligament, obliteration of the angle of His and an enlargement of the crural orifice disrupt the sphincter mechanism. A large paraesophageal hernial component can also compress the lower esophagus, thus exacerbating even further those symptoms. We however note that LHH do not typically present with significant reflux as do smaller ones.

Patients with large herniae often present with other atypical symptoms. Some can develop symptomatic iron deficiency anaemia secondary to chronic blood loss from Cameron’s ulcers. These are mucosal ulcerations which result from pressure effect of the esophageal diaphragmatic hiatus onto the stomach. However, all PEH patients with anaemia should be investigated for other causes of blood loss with colonoscopy and pill cam study in selected cases.

Breathlessness or dyspnoea can be a presenting symptom of such large herniae due to direct mechanical effect on the lung, ventilation/perfusion mismatch and aspirations. A study on 30 patients with giant hiatal herniae showed resolution of preoperative shortness of breath after repair [15]. An improvement of FEV1 and FVC by about 20% was noted by Low et al. in their study on intrathoratic herniae, with two patients even discontinuing home oxygen post surgery [16]. Patients need to be evaluated very carefully and operation not offered simply to improve the lung function if patient is not overtly dyspnoeic.

Patients can also present with syncopal episodes or dizziness due to cardiac compromise from mass effect of the hernia on the heart. Recent investigations have also shown post-prandial reduction in ejection fraction and right atrial/ventricular volume in patients with > 30% intrathoracic hernia. This effect is reversed post operatively with a corresponding improvement in exercise tolerance [17, 18]. Such demonstration of cardiac inflow compromise needs to be carefully undertaken and echocardiography performed before cardiac compromise can be ascribed to the hernia. Other potentially alarming symptoms in the context of LHH include persistent epigastric and chest pain, recurring regurgitation and vomiting, and severe dysphagia to solid food and liquids.

### Timing of surgery for acutely symptomatic patients: acute if you must, subacute staged approach if you can, but avoid prolonged delayed repair

Patients with large hiatus hernia who present acutely need careful assessment. If they are fit to undergo an operation, then it needs to be offered. There is evidence to show that a simple conservative approach in such symptomatic patients has a risk of mortality of 16% [19]. However, there is also a 16% risk of death associated with acute intervention. This is due to the significant complications that those patients present with (perforation with mediastinitis, aspiration), which leads to significant physiological compromise peri operatively. In addition, these patients are generally elderly with significant comorbidities. So, is there a way of improving on the mortality of those who need urgent intervention? There may be a role for a subacute staged approach for those who are not in an immediate organ threatening situation. Two series looking at those who present acutely with large hiatal herniae found that only less than 12.5% required an emergency operation, with the rest being able to proceed to semi-elective or elective repair with no increase in mortality [20, 21]. The delayed strategy includes decompression, restoration of physiological deficits, institution of enteral nutrition, careful anaesthetic evaluation and referring the patient to an experienced team of laparoscopic surgeons for the repair. Interrogation of the NSQIP database however suggests that the delay should be used for patient optimisation but that a prolonged delay will impact adversely on patient outcome [22].

### Can we operate on elderly patients? The risks are high and the key is careful selection

There are a few factors intrinsic to laparoscopic LHH repair that make the endeavour fraught with risks. The operation itself can be prolonged, which in turn can have adverse effect on respiratory and cardiovascular system of those patients who often have underlying co-morbidities such as obstructive airways disease or cardiomyopathy. Mediastinal dissection with the risk of pleural breach causing a pneumothorax or pericardial injury can be poorly tolerated intra- or post-operatively.

A nationwide audit in the US showed an increased mortality rate of 15.6% when repair was performed in octogenareans. Another retrospective study comparing outcomes in age groups (< 69, 70–79 and > 80) found not difference in mortality but showed that 13.3% of octogenareans required prolonged intubation and ICU stay [23]. Interestingly, an assessment of the NSQIP database showed that it is not the absolute age but rather the frailty score, or physiological frailty, which is associated with increased complication and mortality rates [24].

Unsurprisingly, Oor et al. demonstrated that a strategy of operating on well selected patients can lead to a good outcome irrespective of age. There was no 30-day mortality in patients either over or below 70 years old who underwent repair of LHH. The only difference was that elderly patients had a longer length of hospital stay [25].

### Shrinking left lateral lobe can improve hiatal exposure

The left lateral lobe overhangs the esophageal hiatus and requires mechanical retraction to enable safe hiatal hernia repair. The presence of an enlarged fatty left lobe makes the repair of a LHH even more challenging and hazardous. Firstly, the prolonged retraction of the enlarged left lateral lobe can lead to injury and ischemia. Secondly, it obscures the large hiatus and right crus. Dissection becomes more difficult with higher risk of inadvertent trauma to the liver. It also compromises the view required for safe mediastinal dissection, especially should the pneumoperitoneum need to be reduced or in the setting of a pneumothorax. The repair phase is also harder especially whilst suturing of the stomach to the diaphragm and right crus during a Dor anterior fundoplication.

In anticipation of this problem the senior author assesses the size of the left lateral lobe with CT-imaging, especially in patients with BMI > 30 who are at risk of liver steatosis. In case of hepatomegaly the patient is given a very-low-calorie replacement diet of Optifast for one or two weeks preoperatively, which has been associated with significant left lobe shrinkage in the author’s experience.

## Large hiatal herniae require a different evaluation strategy

Although the investigative armamentarium at the disposal of the surgeon is the same for small or large hiatal herniae, the latter require a different approach. The aim is to assess for anatomical rather than functional issues.

Luminal anatomy is assessed with a gastroscopy. The length of the esophagus is measured by assessing the position of the gastroesophageal junction with respect to the crural impression. This is often inaccurate due to anatomical contortion and lack of appreciation of the elasticity of the esophagus, which is best noted intraoperatively. The mucosa is assessed for Barrett’s changes, which can be present in up to 13% of those patients. Strictures and esophagitis are also noted as they may predict the presence of a shortened esophagus. Cameron’s ulcers are looked for, usually at the level of the crura which causes pressure related mucosal ulcerations. In the acute setting gastroscopy is useful to assess mucosal viability in equivocal cases, and help inserting a feeding nasojejunal tube in case a staged delayed approach to repair is entertained.

Extraluminal anatomy is best assessed with an upper abdominal/chest CT with oral and iv contrast. The extent of mediastinal involvement is appreciated on the coronal slices. The size of the hernial sac, cranio-caudal extent of the hernia and relationship to the pericardium and lungs can be objectively appreciated and calculated. The contents of the sac are also noted and would commonly contain the stomach and omentum. The transverse colon can be present which poses no great issue. However, the presence of the spleen, tail of pancreas or even the left lateral lobe of the liver increase the complexity of the operation and may well require a multidisciplinary approach to management. The location of the splanchnic vessels, especially left gastric and splenic arteries, should be carefully noted as any distortion of the anatomy could cause inadvertent injury especially during posterior dissection at the level of the crura. The axial view of the abdominal CT is also useful in assessing intercrural distance, which would predict the likelihood of needing mesh reinforcement. In the acute setting an emergency CT assesses for volvulus, ischemia and perforation.

Functional studies such as manometry and pH studies have the theoretical benefits of confirming reflux and excluding motility disorders. However, they are mainly useful in work up of small hiatal herniae. In more than 50% of the time the probes are not able to negotiate the distorted anatomy of the gastroesophageal junction. Also, the senior author usually performs an anterior Dor fundoplication, which does not compromise those patients who may have underlying undiagnosed esophageal motility disorders.

Extra-gastrointestinal tests may be indicated in selected patients. A preoperative cardiac echo is performed with a history of syncope or dizziness, or in the presence of already known cardiac condition. This will allow direct comparison with post-operative echocardiogram. The latter is always performed to document the absence of pericardial effusion/haematoma secondary to mediastinal dissection. Lung function tests (FEV1, FVC) is also relevant in those who may develop dyspnoea secondary to the compressive effect of the hernia.

## Need for good technique of dissection

### Who should operate?

A recent cohort study has shown that those LHHs are usually present in older patients, have significantly larger defects and carry a higher risk of intra- and post-operative complications, compared to small herniae [26]. Hence, there is a need for such cases to be centralised in dedicated units with intensive care facility and undertaken by experienced laparoscopic surgeons, as there is clear correlation between surgeon volume and patient outcome. Furthermore, the conversion rate in specialised centres remains low at around 1.5%. This in itself can also improve morbidity, post-operative pain and length-of-stay [27, 28]. A survey of cases from the National Inpatient Sample in the US has shown that surgeon volume of > 20 cases per year leads to reduced surgical and medical complications [29].

The benefit of laparoscopic approach by an experienced surgeon extends to the follow up period with a recognised reduced long-term recurrence rate [30]. Functional outcome also improves with dysphagia rate decreasing from 22 to 4%, once the surgeon has performed more than 100 cases [31].

### Prevention and management of intraoperative pneumothorax

Mediastinal dissection for LHH is likely to cause pleural breach and pneumothorax, that may significantly affect ventilation/oxygenation although its magnitude cannot be predicted. It should be avoided, especially in patients with concomitant lung disease. If left unrecognised this could lead to hypotension from reduced venous return with the need for inotropic support. This may also have serious adverse effect in those with cerebrovascular disease or mesenteric vascular disease.

Falk suggests that prevention is the key. Careful mediastinal dissection is required with sweeping motion performed towards the lung. Should there be a pleural breach it should be recognised and dealt with promptly. The pleural defect can be endolooped, sutured or clipped [32]. At any rate, with modern surgical and anaesthetic techniques it is rare to convert to open purely for an intraoperative pneumothorax. The incidence of a pneumothorax is often underreported as the lung generally rapidly re-expands postoperatively. In a series of fundoplications for intrathoracic herniae, 6.25% of patients were noted to have symptomatic pneumothorax [33]. In case of recognised pleural breach during surgery, the author will routinely request a chest Xray in recovery to confirm complete lung re-expansion. Also generally unnecessary, insertion of a pleural drain may be useful in the presence of a persistent pneumothorax.

### Need for hernial sac dissection

In 1999 Watson et al. reported an ‘extrasaccular’ approach to mediastinal dissection of a large hiatal hernia [34]. They penetrated the sac close to the edge of the hiatal defect and then entered the mediastinal areolar plane before bringing the whole sac and its contents back into the abdominal cavity. This avoided traumatic manipulation and injuries to the stomach. A stomach first approach is more difficult especially as it is adherent to the sac posteriorly. Such an approach would also be associated with an increased risk of vagal injuries. They found that the conversion rate to open was reduced from 40 to 9% with this technique.

Complete hernial sac dissection and excision is necessary but could theoretically lead to distal esophageal devascularization and potentially leaks. In fact, in a series on LHHs there were only two leaks noted in 131 patients. Those leaks were delayed (at 7 and 18 days postoperative), which makes them more likely to have been secondary to esophageal suturing performed during the Toupet fundoplication [35]. Whilst sac excision is preferable, the extent of its dissection is dependent on the likelihood of a safe and uncomplicated excision, indication for surgery and skillset of the surgeon.

### Collis gastroplasty: best to avoid

Collis gastroplasty (CG) is performed in the setting of a LHH repair whenever a shortened esophagus (SE) is diagnosed. This entity has been defined by Barrett in 1950 as a situation where the esophageal length is insufficient to allow the gastroesophageal junction (GEJ) to lie below the diaphragm by 2–3 cm.

There are some conditions that predispose to the shortened esophagus such as long esophageal stricture, extensive Barrett’s changes, or grade 3 or 4 esophagitis. These have been mostly prevented by the widespread use of proton pump inhibitors prior to hiatal repair. However, a review of the literature as shown that the technique is employed more than expected and there is great variability in reporting of CG, ranging from 0 to 80% [10, 36, 37]. Even across series from the same institutions the rate of Collis has varied in time. Thus, Luketich et al. reported a decrease in rate from 86 to 53% [38] whereas Zehetner et al. showed an increase in rate of uptake of the technique from 0 to 40% [39]. Such inconsistency suggests that CG is perhaps being done for the wrong indication.

Why is there such variability in uptake of that technique? This is because SE is being over-diagnosed. Esophageal shortening is not a single entity but rather a conglomerate of 3 subtypes as defined by Horvath [40]. Apart from the rarer true non reducible SE there are 2 more common situations encountered. There could be an ‘apparent SE’, such as in large intrathoracic herniae when in fact at the time of the operation the GEJ can be repositioned intraabdominally following adequate esophageal mobilisation. Then there is the ‘true reducible SE’ whereby there ought to be proper full mediastinal dissection of the esophagous to enable caudal displacement of the GEJ back below the diaphragm. Often the SE is judged via preoperative investigations such as a barium contrast study, which has a positive predictive value of only 50% for large herniae. Any preconceived expectation of the surgeon can allow an erroneous use of CG when it is not required. In fact, the SE can only reliably be diagnosed intraoperatively. Unfortunately, this assessment is often done suboptimally and for various reasons. There needs to be an aggressive extra-saccular mediastinal dissection before proper evaluation for SE can be undertaken. It can be difficult to assess the true position of the GEJ due to the presence of an unresected fat pad or only partially resected hernial sac. That is why LHHs need to be performed by expert laparoscopic high-volume surgeons who can make a proper intraoperative assessment and hence avoid an unnecessary Collis.

Swanstrom showed 80% of patients with a preoperative possible diagnosis of SE ended up having the GEJ that could actually be mobilised intraabdominally [41] purely by proper dissection technique and expert intraoperative assessment. There are many reasons to be cautious in performing a CG. There is a risk of esophagitis associated with it due to the presence of trapped oxyntic cells in the distal neo-esophagus. The wrap and reduced distal motility of that neo-segment may also give rise to esophagitis and dysphagia. The main risk of the technique is a leak which occurs in 2–7.5% of cases [42]. It is reportedly more common when performed via the abdominal approach due to crossing of the staple lines. There is also the added risk of fundal ischemia when the short gastric vessels are taken. A large series from Pittsburg on giant hiatal hernia repairs showed that 88% of leaks were noted in patients who had undergone a Collis gastroplasty [38]. Such leaks can be life threatening and lead to escalation of surgical management. Morino et al. reported on a patient with a LHH who underwent a Collis gastroplasty that was complicated by delayed perforation and requiring esophagectomy with colonic interposition graft [42].

## Repair technique

### Cruroplasty: where and how?

Cruroplasty is a crucial step in the reconstructive phase of the hiatal hernia repair. Posterior cruroplasty is the traditional method used and involves approximation of the crura behind the esophagus. Sometimes an additional anterior cruroplasty may be required to prevent additional posterior reinforcement from causing sigmoid deformity of the esophagus, thus causing dysphagia. Falk et al. have shown that with time recurrence occurs anteriorly due to deterioration in the strength of the central tendon, hence the importance of the anterior sutures in some cases [6].

Interrupted non absorbable sutures such as Ethibond have been favoured for cruroplasties. More recently some surgeons have experimented with running barbed sutures (V-Loc Covidien or Stratafix Ethicon) with good result [43]. The potential advantage is that the tensile strength afforded by the running suture is spread more evenly across the crural pillars.

However, in the presence of a very wide crural defect it may sometimes not be possible to perform satisfactory cruroplasties due to excess tension after crural apposition. In addition, the quality of the pillars is poor, made of attenuated muscle fibres with very little fascia. In those situations, relaxing lateral incisions to allow better medialization of the crura are often used and the resulting defects reinforced with mesh. This can reduce the tension on the crura by 50%. A right-sided incision should be favoured and if a left-sided one is performed then a permanent mesh should be used as reinforcement [44]. However, the repeated stress from about 20,000 diaphragmatic movements a day can easily disrupt the repair. Hence additional mesh reinforcement is often required.

### Should fundoplication be performed and which one to choose?

The anatomical rationale for the fundoplication is to restore the angle of His and hence act as a gastropexy. The added value of fundoplication is reduction of the risk of reflux that occurs after full disruption of the GOJ complex that occurs in the process of hiatal dissection. This said, most patients with LHH do not have reflux as the main presenting complaint. Nevertheless, a fundoplication is still advocated in order to reinforce the cruroplasty and thereby aiming at preventing recurrence. Anterior fundoplication such as Dor also anchors the stomach to the diaphragm and crus, thereby preventing the risk of delayed cranial migration of the stomach, unlike simplified Nissen fundoplication. Another benefit of partial wrap is that insertion of a bougie for esophageal calibration is unnecessary, thus avoiding the potential risk of iatrogenic injury.

Up to 65% of patients without an added fundoplication have noted to have reflux post repair. Furnee showed a benefit of having an antireflux procedure in those patients with known reflux disease, with increased normalisation of lower esophageal acid exposure and no increased dysphagia rate [45]. DeMeester even showed a benefit for those with intrathoracic herniae in terms of reduction in acid exposure on pH studies when fundoplication was added, even if the patients had very intermittent marginal reflux symptoms [46].

A recent literature review on LHH looking at mesh vs suture repairs yielded 19 comparative studies. All had undertaken fundoplications, but only 16 published the type used. Eleven had performed a Nissen, six did Toupet and only two did a Dor repair. This preferred choice for LHH is surprising as a Dor type fundoplication with the stomach being secured to the diaphragm and the right crus would be better at preventing wrap migration than a Nissen type repair. Furthermore, the severity of reflux symptoms in this category of LHH is generally low, thus not worrying enough to warrant a full 360 degrees Nissen fundoplication that is not without any unnecessary consequences for the patients such as increased risk of postoperative dysphagia, chest pain, inability to belch or vomit. This might also offer a potential reason for early recurrence post repair. Interestingly Jamieson’s group from Adelaide had shown that a partial anterior fundoplication provides good clinical improvement and patient satisfaction in cases of LHH [47].

‘Telescoping’ is another type of recurrence that has been described. It tends to occur if the esophagus is not secured below the diaphragm. A modified cardiopexy has been described by some authors whereby the cardioesophageal junction and posterior wrap is secured to the median arcuate ligament [48]. Instead, the senior author of this paper performs a routine esophagopexy in addition to an anterior Dor fundoplication.

### Is there a role for PEG or simple gastropexy?

There is no strong evidence regarding the usefulness of any form of simple gastropexy in preventing recurrence in the setting of LHH. A series on large HH repairs showed that all patients who had gastropexy as the only form of treatment were found to have recurrence within a week [49]^.^. A multivariate analysis performed on patients undergoing laparoscopic paraesophageal hernia repairs also found that a simple gastropexy was an independent risk factor for recurrence [50]. Hence there seems to be no role for this as the only treatment during an elective repair of a LHH repair and this simplified technique should be abandoned.

## Role of mesh in large HH repair

### Recurrence rate

Two meta-analysis (MA) in 2016 have shown a benefit of the mesh in reducing post-operative recurrence. Tam et al. showed a 49% reduction in odds of recurrence after mesh reinforcement (OR 0.51, 95% CI 0.25–0.80, p = 0.007) [51]. Huddy et al. found a 64% reduction in odds of recurrence (OR 0.36, 95% CI 0.17–0.77, p = 0.009), although the heterogeneity level was much higher in this study [1]. We note that both MAs considered recurrence when > 2 cm in size. They also included RCTs and comparative cohort studies in their analysis. Conversely, in a more recent study Menon et al. undertook a MA with only previously published RCTs and showed the benefit of the mesh did not reach statistical significance (OR 0.65, 95% CI 0.5–5.41, p = 0.31) [52]. This MA included 5 RCTs with only one having a long enough follow up of 5 years [53] and one being appropriately powered to look for difference in outcome between mesh and suture repair groups [54].

Does the choice of mesh [PTFE, polypropylene (PP), SIS] make any difference? Menon showed a non-significant benefit of synthetic meshes (PTFE and PP) in recurrence prevention [52]. However, Huddy demonstrated a significant benefit of synthetic meshes (PTFE, PP and PTFE/PP), with OR 0.30, 95% CI 0.12–0.73, p = 0.008. We note that none of those two reviews had looked at those particular types of meshes separately. Two studies [55, 56] have shown a significant benefit of PP in reducing hernia recurrence, but this was not confirmed by others [9, 49, 54, 57, 58]. Frantzides’s RCT could demonstrate a significant benefit of PTFE in reducing recurrence after hiatal reinforcement although the study had recruited patients from 1991 to 2000 when LHH repair was at in its infancy and hence a non-reinforced large defect was at greater risk of recurrence [59].

Oeschlager and Watson looked at the potential long-term (up to 5 years) benefit of biological small intestinal submucosa (SIS) mesh and produced conflicting results [53, 54]. Oeschlager showed a non-significant benefit of SIS in reducing recurrence, whilst Watson showed a non-significant benefit of suture repair over SIS. Both of those studies looked at recurrence > 2 cm. So, whilst there seems to be some benefit yielded by mesh reinforcement during hiatal repair, the potentially most promising prosthetic material remains unknown. This said, we believe that a new range of absorbable meshes such as Phasix™ have a promising future, as already demonstrated in the short-term follow-up [60].

### Reoperation rate

Based on 4 RCTs, Memon et al. have shown a three times significantly increased risk of having reoperation with suture repair compared to mesh reinforcement [52]. Similarly, Tam et al. reported a 58% non-significant reduction in odds of reoperation with mesh [51]. We note that reoperation is not always a surrogate of significant hernia recurrence. Reoperations can happen for reasons other than recurrence such as esophageal laceration and mesh erosion. Also, not all patients with significant recurrence may consent for reoperation, mainly due to underlying risks (substantial comorbidities) or if it doesn’t impact negatively on their daily QoL.

Is any type of mesh protective against a reoperation? This has not been well studied by previous meta-analysis. Menon showed a non-significant benefit of non-absorbable meshes against reoperation rates but there was no assessment by specific mesh subtypes [52]. We identified four studies that looked exclusively at the role of PP meshes. Benefit was significant in Grubnik’s study [9], but non-significant in Koetje [57] and Oor’s [58] research. No difference was noted by Watson [54]. Frantzides’s [59] RCT showed a non-significant benefit of PFTE in preventing reoperation, whilst Oeschlager [53] and Watson [54] showed a non-significant benefit with SIS.

In summary, there is currently no categorical significant benefit of mesh usage in reducing reoperation rate. Similarly, there is no mesh type that is of proven benefit in that regard.

### Major intraoperative and mesh related complications

A review of 28 cases of complications from meshes deployed at the hiatus was published by Stadlhuber in 2009 [33]. Seventeen of those patients presented with mesh erosions and nine of them required major foregut resection: six esophagectomies, two partial gastrectomies and one total gastrectomy. Interestingly, whilst 75% of those meshes were synthetic (mainly PTFE and PP), the rest were from biological mesh (mainly Surgisis). A subsequent review a decade later has again confirmed that PTFE and PP are associated with erosions with devastating consequences [91].

Whilst mesh erosions present late, there are other complications that can occur intraoperatively or become obvious in the early post-operative period. These are generally iatrogenic injuries associated with the extensive dissection required for LHH. Leeder reported two esophageal perforations which were repaired intraoperatively. Watson [54] declared two perforations, one from a bougie the other was noted on the third postoperative day. Casteljins reported an intraoperative esophageal perforation that required stenting, with subsequent need for resection and conduit formation [61]. Luckily those complications are not very common. A review of the literature on LHH repair shows that the esophageal perforation and erosion rates were at < 0.5% and < 0.75% respectively. Most patients recovered from the repair.

Is redo surgery worse after mesh? Parker et al. noted that patients who had prior mesh reinforcement had a 6–8 folds increased risk of requiring foregut resection compared to those who had only suture repair at index operation [62]. However, the risk of needing resection increased by four in those with no mesh but who had undergone multiple previous repairs. Interestingly, reoperation in those who had prior biologic mesh reinforcement has been deemed to be easier as the remodelled diaphragm is stronger and represents a better platform to repeat suturing and reinforcement [63].

Does shape of mesh matter? The options are to use a U or a keyhole/circumferential shaped mesh. There are some proponents of the circumferentially deployment as it affords a better contact of the mesh with the surrounding tissue, resulting in perceived improved support. In addition, it allows overlap of the edges of the mesh, hence preventing recurrence as noted in incisional hernia repair [64, 65]. However, this is at the expense of grave risk of mesh erosion as noted as reported in the litterature [66–68]. For instance, Chen who initially placed the mesh in a keyhole manner noted cases of synthetic mesh erosions which all required thoracotomy and mesh removal. Hence the U-shaped deployment is favoured by most centres worldwide. Other techniques to minimise mesh erosions include preserving a cuff of tissue between the mesh and esophagus or using a small piece of a soft large pore mesh [69].

### Need for esophageal dilatation

Post-operative dysphagia is due to a multitude of factors: esophageal dysmotility, tight cruroplasty, sigmoid deformity of the esophagus after repair, 360^°^ fundoplication or fibrosis due to the mesh. Whilst a few studies have reported on patients who required post-operative dilatation for dysphagia, only Ringley compared this rate between mesh and suture groups that was identical at 4.5% (1/22) [70]. This may also indicate that the type of fundoplication (360^0^ Nissen versus partial) probably plays a more important role in the incidence of post-operative stenosis requiring esophageal dilatation [71, 72]. This said, a good result was noted after a single dilatation although follow up period was only 12 months.

In comparison, Bragetto [73], Chen [66], Illyashenko [56] and Leeder [49] reported on overall dilatation rate of 6.2% (5/81), 1.4% (1/69), 1% (1/98) and 2% (1/51) respectively but provided no details in mesh vs suture groups.

### Delayed gastric emptying

Dissection and repair of LHHs carries an increased risk of vagal nerve injury. This could potentially translate into delayed gastric emptying which is often noted post operatively. This phenomenon is also related to old age, diabetes and extended length of time stomach has been incarcerated in the mediastinum. This usually is self-limiting although it can lead to prolonged NGT placement and increased length of stay. This outcome is not always investigated in the literature. In fact, we have found only one comparative study reporting that overall, 2.5% of patients required NGT post-operatively but eventually recovered without intervention [73].

### Mortality

Mortality related to the mesh itself is associated with tacking and mesh erosion. In the acute setting tacking can lead to pericardial tamponade as outlined in Section 7a (ii). A series published on the topic has shown that such tamponades to be fatal in 48% of the cases and that are obvious 1–2 days post operatively [74]. In the long run mesh erosions can lead to the need for foregut resection [33] which has an associated periprocedural mortality, especially as those patients are often older with comorbidities. However, reinterventions are often undertaken in well selected patients and in tertiary referral units. In addition the follow up of those patients is either not well documented or is not undertaken for a long enough period [66, 75, 76]. Hence the reported mortality associated with mesh erosions in the literature is not as high as one would have expected.

### Guidelines

The Society of American Gastroenterological Endoscopic Surgeons (SAGES) guidelines from 2015 [77] is equivocal in its recommendation regarding the role of mesh in hiatal hernia repair, reiterating the lack of strong evidence on its usefulness. However, it recognised that there may be a benefit in mesh reinforcement in LHHs in terms of decreased short term recurrence rate. The European Association of Endoscopic Surgeons (EAES) consensus guideline echoed the guarded suggestion from SAGES but recommending the selective use of the mesh in those patients with large crura and large hiatal defect [78].

There have been two surveys of European surgeons. Huddy et al. noted that 67% of responders used mesh (7% routine, 60% selective), with two thirds of those using synthetic rather than absorbable meshes. This uptake is surprisingly high given that 20% of those surveyed have had personal experience with mesh erosion in their practice [1]. Furnee surveyed European surgeons with regards to LHH repair [2]. 14.5% used mesh routinely and 77.6% were selective users. 52.6% used polypropylene mesh. A survey of American surgeons in 2007 showed that 10% used mesh routinely with 46% favouring synthetic mesh (polypropylene and PTFE) and 28% using biomaterial [79]. NSQIP database in period 2011–14 showed a 39% mesh uptake in laparoscopic paraesophageal hernia repairs [29].

### So, to mesh or not to mesh LHH?

Mesh reduces total recurrence significantly with a lesser impact on reoperation rate. The relative benefit of PFTE/PP/SIS has not been proven on comparative studies. Overall, the mortality and major morbidity of LHH mesh repair is acceptable. Although rare, the risk of erosion from synthetic mesh is concerning and it is now time to consider other alternative. A close look at long-term study with synthetic and biological meshes suggests that the future may lie with biosynthetic meshes (BSMs).

Table 1 summarises the timing of publication (comparative, cohort studies) of meshes (synthetic, biological, biosynthetic) used in LHH. The evidence on BSM is dominated by absorbable BioA, with four cohort and only one comparative study. New data on the more recent BSM of Phasix ST is now emerging, with a recent cohort study on LHH showing promising result, although follow up was short [60]. Phasix ST has an improved profile compared to BioA as it handles better and is reabsorbed within 18 months, versus 6 months. The construction of well powered RCTs involving biosynthetics with long term follow up and paying particular attention to standardization of variables (such as type of hernia, surgical techniques) will hopefully determine if newer BSMs are in fact the answer we have been looking for.

## Post op care

### Acute inpatient care

#### Role of postoperative contrast study

In surgical practice the use of contrast swallow study in the immediate post-operative setting is variable. Some units prefer to do it routinely whilst others do it ‘on demand’ depending on the patient’s symptoms and progress. The rationale for a gastrograffin study would be mainly to exclude the diagnosis of an early iatrogenic esophageal perforation, before resuming oral intake. Likewise, the reason to opt for an immediate post-operative barium swallow study would be mainly to confirm success of the repair (formally excluding primary failed anti-reflux surgery), in the context of a clinical study or RCT. For instance, Watson et al. [80] performed barium meals routinely in all the patients enrolled in their randomised controlled trial. Out of the 126 patients, 13 ended up having reoperations. Of those, 6 (4.8%) had early repairs: 3 had a tight GEJ and 3 had acute herniations. It was hard to know which ones were subclinical and had repair before they became symptomatic. The same group found that actually 0.8% (1 in 125) benefitted from early post-operative contrast studies and resulted in an early reoperation [81].

In our institution we do not perform contrast studies routinely in the immediate post-operative period after LHH repairs. Our aim is to mainly focus on a swift post-operative recovery as these patients are often old and frail, rather than actively looking for a potentially small residual hernia that would generally not warrant any immediate or even delayed surgical correction. Instead, we offer a routine endoscopy at 4 months and one year post surgery that offers, in our experience, a better alternative.

#### Role of cardiac echocardiogram

Although very rare this is a much dreaded almost fatal complication after LHH repair. It can arise after extensive dissection around the pericardium. An unrecognised inadvertent injury can lead to a haemopericardium. Alternatively, it can result in an ‘early pericardiotomy syndrome’ where a local inflammatory reaction can result in accumulation of serous fluid in the pericardial sac [82]. It can also arise as a result of mechanical related injuries. A review by Kockerling [74] revealed 25 cases of cardiac tamponade directly related to mesh fixation with either sutures or tacks, and leading to injury of the pericardium, coronary artery or vein, or epicardial artery. Such injuries with tacks are predictable given the depth of penetration (3.7–5.9 mm) relative to the diaphragmatic tendon (3 mm). The hemopericardium was usually clinically obvious by day 1-2 and fatal in 48% of the reported cases in the review mentioned.

Hence, the senior author never fixes the mesh with tacks near the diaphragm. Instead, the prosthesis is only secured with fibrin glue (Tisseel). Careful suturing is exclusively performed during the anterior partial Dor fundoplication, close to the repaired esophageal hiatus. More importantly, we routinely admit postoperative extubated patients to the high dependency unit where they undergo invasive 24 hours monitoring. A cardiac echocardiogram is then routinely performed to rule out a subclinical effusion/haemopericardium before it becomes symptomatic.

### Long term follow up: selective or routine?

Patients undergoing repair for small symptomatic hiatal hernia are often young and are followed up regularly for long period of time due to the risk of recurrence during their lifetime. In comparison, the recurrence rate for LHH is even higher, ranging between 15 and 40% [30, 83]. However, the data used to document the laparoscopic recurrences for LHH repair came from studies published in 2000-2005, with the surgeries being performed in the 1990s when laparoscopic technique was at its infancy. Also, there is a paucity of long term follow up studies on this topic. Even among those studies the proportion of patients having regular objective assessment was variable. Hence, the true recurrence rate still remains unknown. Moreover, only 5% have been known to have symptomatic recurrences [57].

So, should we routinely follow up patients after LHH repair? There are a few reasons to suggest a more selective approach. Firstly, those patients are already 60-70 years old at the time of their first repair. A follow up strategy centred around symptoms in those who are physically fit would make more sense as those patients may potentially well benefit from a reoperation should they subsequently develop clinically significant recurrence. Secondly, those patients usually undergo their first operation for complications related to the sheer size of their hernia i.e. risk of volvulus, iron deficiency anaemia from Cameron’s ulcers or cardiopulmonary compression. Therefore, a strict follow up strategy aiming at finding small recurrences with time does not really apply to them. Thirdly, QoL seems to be generally preserved despite the advent of recurrences. This is substantiated by others who showed that an aggressive post-operative follow up regime with the main goal of assessing for objective recurrence but without proper evaluation of the subjective assessment of the patient (i.e. symptom resolution, satisfaction, well-being and QoL) was not useful [84, 85].

We would therefore prefer to adopt a more selective approach in following up patients postoperatively with the focus being on recurrence of symptoms rather than hernia recurrence. Patients should be followed up clinically yearly until they are deemed unfit for reintervention. In that interval, should they develop significant symptoms repeat gastroscopy with or without upper abdominal/chest CT scan or barium fluoroscopic study may be necessary to objectively assess for recurrence.

## Mortality related to repair

A literature survey on LHH performed over the last 3 decades has shown that overall 30-day post-operative mortality was < 1%. There are a number of factors associated with mortality in LHH repair. Firstly, it could be related medical complications. Thus there are reports of postoperative death from myocardial infarction and pulmonary emboli that were not considered as direct surgical but medical complications [53, 54]. The rate of mortality from such medical complications is not very high which is probably a reflection of improvement in patient selection, anaesthesia and post-operative care. Secondly mortality can be related to surgical complications as they are not well tolerated in this group of elderly patients. For example, we note the case of a 75-year-old who developed a gastric perforation post-surgical repair of an intrathoracic stomach. He succumbed to a fatal gastropleural fistula 2 months later [49]. Thirdly they could occur intraoperatively from aortic injury especially due to adhesions and anatomical distortion caused by the LHH [61]. They are fortunately a rare occurrence. Forth, the mortality is significantly increased in emergency cases. An assessment of 10656 cases from the National Surgical Quality Improvement Program database showed a 30day mortality of 5.5% in acute cases and 0.65% in elective cases [86]. A logistic regression analysis identified acute surgery together with old age and cardiopulmonary disease to be predictors of 30 day mortality [87]

## Role of robotic repair

The robotic platform has some inherent characteristics that are well suited for large hiatus hernia surgery. The length and increased degree of freedom of the robotic instruments make manoeuvrability and dissection in the narrow corridor of the esophageal hiatus and mediastinum potentially easier. The 3D visualisation afforded by the camera which is controlled by the surgeon’s console gives a better depth perception in a hostile environment where dissection planes dictate the extent of intraoperative complications. In addition, tremor filtration, motion scaling and better ergonomics associated with the robotic surgery improve the surgeon’s efficiency especially given that those operations are lengthy.

However, there are still three areas that need development before robotic surgery can outperform laparoscopic repair. Firstly, use of the robot adds more time to an already difficult repair. A meta analysis comparing robot assisted repair and conventional laparoscopic technique found the mean dissection time with the robot to be significantly longer [88]. Secondly there is the inherently higher cost of robotic surgery. A recent comparative retrospective series showed charge of a robotic case to be US$15-20,000 higher than for laparoscopy [89]. Thirdly and most importantly the loss of haptic feedback with the robot can lead to increased complications. Increased risk of respiratory failure and esophageal/gastric perforations can occur as a result of poor tissue handling and tearing [89, 90]. However improved design of subsequent generations of the robot, early incorporation of robotic modules into surgical training and widespread accessibility of the robot will eventually make it a better competitor to the laparoscopic technique.

## Conclusion

In summary, we suggest a careful evaluation and selection of all patients with a LHH. The latter should be evaluated endoscopically and with a CT chest. Those with a large fatty left lateral lobe should undergo a routine preoperative optifast regime. We undertake a careful and meticulous surgical approach to such patients. This includes systematic excision of the hernia sac and extensive mediastinal extra-saccular dissection to allow full mobilisation of the GEJ back into the abdomen, thus avoiding an unnecessary Collis gastroplasty, which has associated morbidity. We advise posterior cruroplasty with biosynthetic mesh reinforcement in a U-shaped configuration, away from the esophagus. The prosthesis is then preferentially fixed with non-traumatic fibrin glue. We favour an anterior partial Dor fundoplication with esophagopexy. We recommend that all patients undergo a cardiac echo the next day. Upon discharge a selective follow up regimen is put in place.

**Table 1:**
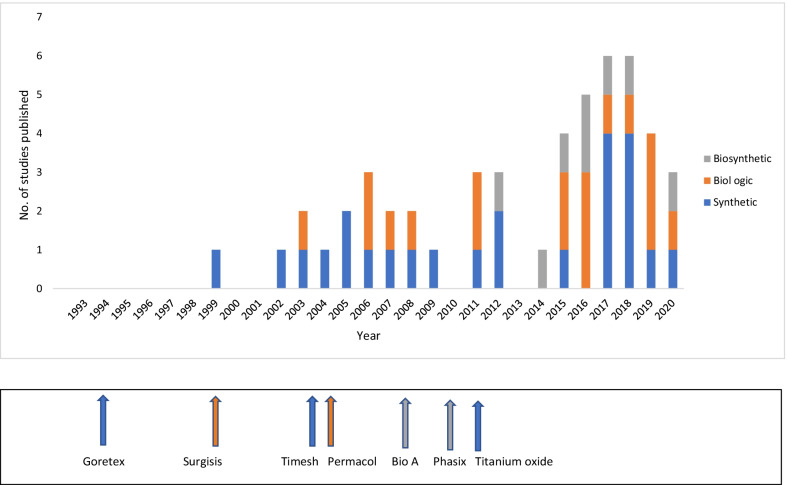
Showing frequency of publication of studies on different types of mesh each year

The year new types of mesh have been out on the market has been highlighted

## Data Availability

Not applicable.

## Abbreviations

LHH: Large hiatus hernia
HH: Hiatus Hernia
PEH: ParaEsophageal hernia
NSQIP: National Surgical Quality Improvement
FEV1: Forced expired volume
FVC: Forced vital capacity
CG: Collis Gastroplasty
SE: Shortened esophagus
GOJ: GastroOesophageal junction
MA: Meta analysis
PTFE: PolyTetraFluoroEthylene
PP: Polypropylene
SIS: Surgisis

## References

[1] Huddy JR, Markar SR, Ni MZ, Morino M, Targarona EM, Zaninotto G, Hanna GB (2016). Laparoscopic repair of hiatus hernia: Does mesh type influence outcome? A meta-analysis and European survey study. Surg Endosc.

[2] Furnée EJ, Smith CD, Hazebroek EJ (2015). The use of mesh in laparoscopic large hiatal hernia repair: a survey of european surgeons. Surg Laparosc Endosc Percutan Tech.

[3] Jay AP, Watson DI (2010). Changing work patterns for benign upper gastrointestinal and biliary disease: 1994–2007. ANZ J Surg.

[4] Reynolds JL, Zehetner J, Bildzukewicz N, Katkhouda N, Lipham JC (2016). A durable laparoscopic technique for the repair of large paraesophageal hernias. Am Surg.

[5] Mitiek MO, Andrade RS (2010). Giant hiatal hernia. Ann Thorac Surg.

[6] Furtado RV, Vivian SJ, van der Wall H, Falk GL (2016). Medium-term durability of giant hiatus hernia repair without mesh. Ann R Coll Surg Engl.

[7] Champion JK, Rock D (2003). Laparoscopic mesh cruroplasty for large paraesophageal hernias. Surg Endosc.

[8] Granderath FA, Schweiger UM, Pointner R (2007). Laparoscopic antireflux surgery: tailoring the hiatal closure to the size of hiatal surface area. Surg Endosc.

[9] Grubnik VV, Malynovskyy AV (2013). Laparoscopic repair of hiatal hernias: new classification supported by long-term results. Surg Endosc.

[10] Maziak DE, Todd TR, Pearson FG (1998). Massive hiatus hernia: evaluation and surgical management. J Thorac Cardiovasc Surg.

[11] Skinner DB, Belsey RH (1967). Surgical management of esophageal reflux and hiatus hernia. Long-term results with 1,030 patients. J Thorac Cardiovasc Surg.

[12] Stylopoulos N, Gazelle GS, Rattner DW (2002). Paraesophageal hernias: operation or observation?. Ann Surg.

[13] Jung JJ, Naimark DM, Behman R, Grantcharov TP (2018). Approach to asymptomatic paraesophageal hernia: watchful waiting or elective laparoscopic hernia repair?. Surg Endosc.

[14] Paul S, Mirza FM, Nasar A, Port JL, Lee PC, Stiles BM, Nguyen AB, Sedrakyan A, Altorki NK (2011). Prevalence, outcomes, and a risk-benefit analysis of diaphragmatic hernia admissions: an examination of the National Inpatient Sample database. J Thorac Cardiovasc Surg.

[15] Zhu JC, Becerril G, Marasovic K, Ing AJ, Falk GL (2011). Laparoscopic repair of large hiatal hernia: impact on dyspnoea. Surg Endosc.

[16] Low DE, Simchuk EJ (2002). Effect of paraesophageal hernia repair on pulmonary function. Ann Thorac Surg.

[17] Milito P, Lombardi M, Asti E, Bonitta G, Fina D, Bandera F, Bonavina L (2018). Influence of large hiatus hernia on cardiac volumes. A prospective observational cohort study by cardiovascular magnetic resonance. Int J Cardiol.

[18] Naoum C, Kritharides L, Falk GL, Martin D, Yiannikas J (2018). Left atrial compression and right ventricular outflow tract diameter on echocardiography are independently associated with exercise capacity in patients with large hiatal hernia. Echocardiography.

[19] Sihvo EI, Salo JA, Räsänen JV, Rantanen TK (2009). Fatal complications of adult paraesophageal hernia: a population-based study. J Thorac Cardiovasc Surg.

[20] Köhler G, Koch OO, Antoniou SA, Emmanuel K, Pointner R (2015). "Acute intrathoracic stomach!" How should we deal with complicated type IV paraesophageal hernias?. Hernia.

[21] Wirsching A, El Lakis MA, Mohiuddin K, Pozzi A, Hubka M, Low DE (2018). Acute vs. elective paraesophageal hernia repair: endoscopic gastric decompression allows semi-elective surgery in a majority of acute patients. J Gastrointest Surg.

[22] Bhayani NH, Kurian AA, Sharata AM, Reavis KM, Dunst CM, Swanstrom LL (2013). Wait only to resuscitate: early surgery for acutely presenting paraesophageal hernias yields better outcomes. Surg Endosc.

[23] Parker DM, Rambhajan AA, Horsley RD, Johanson K, Gabrielsen JD, Petrick AT (2017). Laparoscopic paraesophageal hernia repair is safe in elderly patients. Surg Endosc.

[24] Chimukangara M, Frelich MJ, Bosler ME, Rein LE, Szabo A, Gould JC (2016). The impact of frailty on outcomes of paraesophageal hernia repair. J Surg Res.

[25] Oor JE, Koetje JH, Roks DJ, Nieuwenhuijs VB, Hazebroek EJ (2016). Laparoscopic Hiatal Hernia Repair in the Elderly Patient. World J Surg.

[26] Köckerling F, Trommer Y, Zarras K, Adolf D, Kraft B, Weyhe D, Fortelny R, Schug-Paß C (2017). What are the differences in the outcome of laparoscopic axial (I) versus paraesophageal (II-IV) hiatal hernia repair?. Surg Endosc.

[27] Bàllesta López C, Cid JA, Poves I, Bettónica C, Villegas L, Memon MA (2003). Laparoscopic surgery in the elderly patient. Surg Endosc.

[28] Weber DM (2003). Laparoscopic surgery: an excellent approach in elderly patients. Arch Surg.

[29] Schlottmann F, Strassle PD, Farrell TM, Patti MG (2017). Minimally invasive surgery should be the standard of care for paraesophageal hernia repair. J Gastrointest Surg.

[30] Hashemi M, Peters JH, DeMeester TR, Huprich JE, Quek M, Hagen JA, Crookes PF, Theisen J, DeMeester SR, Sillin LF, Bremner CG (2000). Laparoscopic repair of large type III hiatal hernia: objective followup reveals high recurrence rate. J Am Coll Surg.

[31] Castelijns PS, Ponten JE, Bouvy ND, Smulders JF, van de Poll MC (2018). Intrathoracic stomach in hiatal hernia: the role of laparoscopic repair. Minerva Chir.

[32] Falk GL, D'Netto TJ, Phillips S, Little SC (2018). Pneumothorax: laparoscopic intraoperative management during fundoplication facilitates management of cardiopulmonary instability and surgical exposure. J Laparoendosc Adv Surg Tech A.

[33] Stadlhuber RJ, Sherif AE, Mittal SK, Fitzgibbons RJ, Michael Brunt L, Hunter JG, Demeester TR, Swanstrom LL, Daniel Smith C, Filipi CJ (2009). Mesh complications after prosthetic reinforcement of hiatal closure: a 28-case series. Surg Endosc.

[34] Watson DI, Davies N, Devitt PG, Jamieson GG (1999). Importance of dissection of the hernial sac in laparoscopic surgery for large hiatal hernias. Arch Surg.

[35] Banki F, Kaushik C, Roife D, Mitchell KG, Miller CC (2017). Laparoscopic repair of large hiatal hernia without the need for esophageal lengthening with low morbidity and rare symptomatic recurrence. Semin Thorac Cardiovasc Surg.

[36] Jobe BA, Aye RW, Deveney CW, Domreis JS, Hill LD (2002). Laparoscopic management of giant type III hiatal hernia and short esophagus. Objective follow-up at three years. J Gastrointest Surg.

[37] Jones R, Tadaki C, Oleynikov D (2015). Laparoscopic redo paraesophageal hernia repair with collis gastroplasty for shortened esophagus. Surg Endosc.

[38] Luketich JD, Nason KS, Christie NA, Pennathur A, Jobe BA, Landreneau RJ, Schuchert MJ (2010). Outcomes after a decade of laparoscopic giant paraesophageal hernia repair. J Thorac Cardiovasc Surg.

[39] Zehetner J, Demeester SR, Ayazi S, Kilday P, Augustin F, Hagen JA, Lipham JC, Sohn HJ, Demeester TR (2011). Laparoscopic versus open repair of paraesophageal hernia: the second decade. J Am Coll Surg.

[40] Horvath KD, Swanstrom LL, Jobe BA (2000). The short esophagus: pathophysiology, incidence, presentation, and treatment in the era of laparoscopic antireflux surgery. Ann Surg.

[41] Swanstrom LL, Marcus DR, Galloway GQ (1996). Laparoscopic Collis gastroplasty is the treatment of choice for the shortened esophagus. Am J Surg.

[42] Morino M, Giaccone C, Pellegrino L, Rebecchi F (2006). Laparoscopic management of giant hiatal hernia: factors influencing long-term outcome. Surg Endosc.

[43] Wade A, Dugan A, Plymale MA, Hoskins J, Zachem A, Roth JS (2016). Hiatal hernia cruroplasty with a running barbed suture compared to interrupted suture repair. Am Surg.

[44] Crespin OM, Yates RB, Martin AV, Pellegrini CA, Oelschlager BK (2016). The use of crural relaxing incisions with biologic mesh reinforcement during laparoscopic repair of complex hiatal hernias. Surg Endosc.

[45] Furnée EJ, Draaisma WA, Gooszen HG, Hazebroek EJ, Smout AJ, Broeders IA (2011). Tailored or routine addition of an antireflux fundoplication in laparoscopic large hiatal hernia repair: a comparative cohort study. World J Surg.

[46] Fuller CB, Hagen JA, DeMeester TR, Peters JH, Ritter M, Bremmer CG (1996). The role of fundoplication in the treatment of type II paraesophageal hernia. J Thorac Cardiovasc Surg.

[47] Shukri MJ, Watson DI, Lally CJ, Devitt PG, Jamieson GG (2008). Laparoscopic anterior 90 degree fundoplication for reflux or large hiatus hernia. ANZ J Surg.

[48] Suppiah A, Sirimanna P, Vivian SJ, O'Donnell H, Lee G, Falk GL (2017). Temporal patterns of hiatus hernia recurrence and hiatal failure: quality of life and recurrence after revision surgery. Dis Esophagus.

[49] Leeder PC, Smith G, Dehn TC (2003). Laparoscopic management of large paraesophageal hiatal hernia. Surg Endosc.

[50] Larusson HJ, Zingg U, Hahnloser D, Delport K, Seifert B, Oertli D (2009). Predictive factors for morbidity and mortality in patients undergoing laparoscopic paraesophageal hernia repair: age, ASA score and operation type influence morbidity. World J Surg.

[51] Tam V, Winger DG, Nason KS (2016). A systematic review and meta-analysis of mesh vs suture cruroplasty in laparoscopic large hiatal hernia repair. Am J Surg.

[52] Memon MA, Siddaiah-Subramanya M, Yunus RM, Memon B, Khan S (2019). Suture cruroplasty versus mesh hiatal herniorrhaphy for large hiatal hernias (HHs): an updated meta-analysis and systematic review of randomized controlled trials. Surg Laparosc Endosc Percutan Tech.

[53] Oelschlager BK, Pellegrini CA, Hunter JG, Brunt ML, Soper NJ, Sheppard BC, Polissar NL, Neradilek MB, Mitsumori LM, Rohrmann CA, Swanstrom LL (2011). Biologic prosthesis to prevent recurrence after laparoscopic paraesophageal hernia repair: long-term follow-up from a multicenter, prospective, randomized trial. J Am Coll Surg.

[54] Watson DI, Thompson SK, Devitt PG, Aly A, Irvine T, Woods SD, Gan S, Game PA, Jamieson GG (2020). Five year follow-up of a randomized controlled trial of laparoscopic repair of very large hiatus hernia with sutures versus absorbable versus nonabsorbable mesh. Ann Surg.

[55] Granderath FA, Schweiger UM, Kamolz T, Asche KU, Pointner R (2005). Laparoscopic Nissen fundoplication with prosthetic hiatal closure reduces postoperative intrathoracic wrap herniation: preliminary results of a prospective randomized functional and clinical study. Arch Surg.

[56] Ilyashenko VV, Grubnyk VV, Grubnik VV (2018). Laparoscopic management of large hiatal hernia: mesh method with the use of ProGrip mesh versus standard crural repair. Surg Endosc.

[57] Koetje JH, Oor JE, Roks DJ, Van Westreenen HL, Hazebroek EJ, Nieuwenhuijs VB (2017). Equal patient satisfaction, quality of life and objective recurrence rate after laparoscopic hiatal hernia repair with and without mesh. Surg Endosc.

[58] Oor JE, Roks DJ, Koetje JH, Broeders JA, van Westreenen HL, Nieuwenhuijs VB, Hazebroek EJ (2018). Randomized clinical trial comparing laparoscopic hiatal hernia repair using sutures versus sutures reinforced with non-absorbable mesh. Surg Endosc.

[59] Frantzides CT, Madan AK, Carlson MA, Stavropoulos GP (2002). A prospective, randomized trial of laparoscopic polytetrafluoroethylene (PTFE) patch repair vs simple cruroplasty for large hiatal hernia. Arch Surg.

[60] PaniciTonucci T, Asti E, Sironi A, Ferrari D, Bonavina L (2020). Safety and Efficacy of Crura Augmentation with Phasix ST Mesh for Large Hiatal Hernia: 3-Year Single-Center Experience. J Laparoendosc Adv Surg Tech A.

[61] Castelijns PSS, Ponten JEH, Van de Poll MCG, Nienhuijs SW, Smulders JF (2017). Subjective outcome after laparoscopic hiatal hernia repair for intrathoracic stomach. Langenbecks Arch Surg.

[62] Parker M, Bowers SP, Bray JM, Harris AS, Belli EV, Pfluke JM, Preissler S, Asbun HJ, Smith CD (2010). Hiatal mesh is associated with major resection at revisional operation. Surg Endosc.

[63] Latzko M, Borao F, Squillaro A, Mansson J, Barker W, Baker T (2014). Laparoscopic repair of paraesophageal hernias. JSLS..

[64] Tulloh B, de Beaux A (2016). Defects and donuts: the importance of the mesh:defect area ratio. Hernia.

[65] Binnebösel M, Rosch R, Junge K, Flanagan TC, Schwab R, Schumpelick V, Klinge U (2007). Biomechanical analyses of overlap and mesh dislocation in an incisional hernia model in vitro. Surgery.

[66] Chen Z, Zhao H, Sun X, Wang Z (2018). Laparoscopic repair of large hiatal hernias: clinical outcomes of 10 years. ANZ J Surg.

[67] Yatabe K, Ozawa S, Ito E, Oguma J, Kazuno A, Nitta M, Ninomiya Y (2017). Late esophageal wall injury after mesh repair for large esophageal hiatal hernia: a case report. Surg Case Rep.

[68] Kepenekci I, Turkcapar AG (2009). Mesh erosion as a complication of laparoscopic fundoplication with prosthetic hiatal closure: report of a case. Surg Laparosc Endosc Percutan Tech.

[69] Gordon AC, Gillespie C, Son J, Polhill T, Leibman S, Smith GS (2018). Long-term outcomes of laparoscopic large hiatus hernia repair with nonabsorbable mesh. Dis Esophagus.

[70] Ringley CD, Bochkarev V, Ahmed SI, Vitamvas ML, Oleynikov D (2006). Laparoscopic hiatal hernia repair with human acellular dermal matrix patch: our initial experience. Am J Surg.

[71] Watson DI, Jamieson GG, Lally C, Archer S, Bessell JR, Booth M, Cade R, Cullingford G, Devitt PG, Fletcher DR, Hurley J, Kiroff G, Martin CJ, Martin IJ, Nathanson LK, Windsor JA, International Society for Diseases of the Esophagus-Australasian Section (2004). Multicenter, prospective, double-blind, randomized trial of laparoscopic nissen vs anterior 90 degrees partial fundoplication. Arch Surg.

[72] Trepanier M, Dumitra T, Sorial R, Siblini A, Vassiliou M, Fried GM, Feldman LS, Ferri LE, Lee L, Mueller CL (2019). Comparison of Dor and Nissen fundoplication after laparoscopic paraesophageal hernia repair. Surgery.

[73] Braghetto I, Korn O, Csendes A, Burdiles P, Valladares H, Brunet L (2010). Postoperative results after laparoscopic approach for treatment of large hiatal hernias: is mesh always needed? Is the addition of an antireflux procedure necessary?. Int Surg.

[74] Köckerling F, Schug-Pass C, Bittner R (2018). A word of caution: never use tacks for mesh fixation to the diaphragm!. Surg Endosc.

[75] Gouvas N, Tsiaoussis J, Athanasakis E, Zervakis N, Pechlivanides G, Xynos E (2011). Simple suture or prosthesis hiatal closure in laparoscopic repair of paraesophageal hernia: a retrospective cohort study. Dis Esophagus.

[76] Zaninotto G, Portale G, Costantini M, Fiamingo P, Rampado S, Guirroli E, Nicoletti L, Ancona E (2007). Objective follow-up after laparoscopic repair of large type III hiatal hernia. Assessment of safety and durability. World J Surg.

[77] https://www.sages.org/publications/guidelines/guidelines-for-the-management-of-hiatal-hernia/

[78] Fuchs KH, Babic B, Breithaupt W, Dallemagne B, Fingerhut A, Furnee E, Granderath F, Horvath P, Kardos P, Pointner R, Savarino E, Van Herwaarden-Lindeboom M, Zaninotto G, European Association of Endoscopic Surgery (EAES) (2014). EAES recommendations for the management of gastroesophageal reflux disease. Surg Endosc.

[79] Frantzides CT, Carlson MA, Loizides S, Papafili A, Luu M, Roberts J, Zeni T, Frantzides A (2010). Hiatal hernia repair with mesh: a survey of SAGES members. Surg Endosc.

[80] Watson DI, Thompson SK, Devitt PG, Smith L, Woods SD, Aly A, Gan S, Game PA, Jamieson GG (2015). Laparoscopic repair of very large hiatus hernia with sutures versus absorbable mesh versus nonabsorbable mesh: a randomized controlled trial. Ann Surg.

[81] Tsunoda S, Jamieson GG, Devitt PG, Watson DI, Thompson SK (2010). Early reoperation after laparoscopic fundoplication: the importance of routine postoperative contrast studies. World J Surg.

[82] Makey IA, Beers K, Eilers A, Thomas M (2018). Acute serous tamponade after paraesophageal hernia repair reoperation. BMJ Case Rep.

[83] Aly A, Munt J, Jamieson GG, Ludemann R, Devitt PG, Watson DI (2005). Laparoscopic repair of large hiatal hernias. Br J Surg.

[84] Korolija D, Sauerland S, Wood-Dauphinée S, Abbou CC, Eypasch E, Caballero MG, Lumsden MA, Millat B, Monson JR, Nilsson G, Pointner R, Schwenk W, Shamiyeh A, Szold A, Targarona E, Ure B, Neugebauer E, European Association for Endoscopic Surgery (2004). Evaluation of quality of life after laparoscopic surgery: evidence-based guidelines of the European Association for Endoscopic Surgery. Surg Endosc.

[85] Granderath FA, Carlson MA, Champion JK, Szold A, Basso N, Pointner R, Frantzides CT (2006). Prosthetic closure of the esophageal hiatus in large hiatal hernia repair and laparoscopic antireflux surgery. Surg Endosc.

[86] Kaplan JA, Schecter S, Lin MY, Rogers SJ, Carter JT (2015). Morbidity and mortality associated with elective or emergency paraesophageal hernia repair. JAMA Surg.

[87] Ballian N, Luketich JD, Levy RM, Awais O, Winger D, Weksler B, Landreneau RJ, Nason KS (2013). A clinical prediction rule for perioperative mortality and major morbidity after laparoscopic giant paraesophageal hernia repair. J Thorac Cardiovasc Surg.

[88] Wang Z, Zheng Q, Jin Z (2012). Meta-analysis of robot-assisted versus conventional laparoscopic Nissen fundoplication for gastro-oesophageal reflux disease. ANZ J Surg.

[89] Ward MA, Hasan SS, Sanchez CE, Whitfield EP, Ogola GO, Leeds SG (2021). Complications following robotic hiatal hernia repair are higher compared to laparoscopy. J Gastrointest Surg.

[90] Arcerito M, Perez MG, Kaur H, Annoreno KM, Moon JT (2020). Robotic fundoplication for large paraesophageal hiatal hernias. JSLS..

[91] Li J, Cheng T (2019). Mesh erosion after hiatal hernia repair: the tip of the iceberg?. Hernia.

